# 

**Published:** 1916-07

**Authors:** 


### NEW SUBSCRIBERS

Dr. G. R. Rau------------Ala.
Dr. M. C. Milenden----N. C.
Dr. O. N. Arrington----Miss.
Dr. M. S. Pickard--------La.
Dr. C. T. Nolen----------Ga.
Dr. C. P. Rogers----------Fla.
Dr. T. M. Davis-------S. C.
Dr. M. H. Bell----------Miss.
Dr. Troy C. Sexton----N. Mex.
Dr. J. E. Lanier----------Ga.
Dr. S. A. Pennington------La.
Dr. W. B. Moon-----------Fla.
Dr. Geo. L. Echols--------Ga.
Dr. F. J. Kearny----------La.
Dr. M. H. Hagood--------Ala.
Dr. E. C. Bipus--------W. Va.
Dr. McCurry & McCurry--Ga.
Dr. A. M. Wylie--------S. C.
Dr. Guy B. VanSandt----Okla.
Dr. G. F. Hagood----------Ga.
Dr. B. C. Fry------------La.
Dr. B. S. Burton----------Ga.
Dr. W. R. Groover--------Fla.
Dr. M. B. Herlong---------Fla.
Dr. M. Armstrong------Texas
Dr. G. F. Grisinger----W. Va.

Dr. Paul G. Gates---------Ga.
Dr. James Bennitt------Miss.
C. F. Sayles------------Fla.
M. A. Clark-------------Ga.
H. B. Best------------N. C.
H. J. Erwin------------N. C.
E. W. Bitzer------------Fla.
J. T. McMorele---------Texas
H. H. Gloover------------Ga.
Wm. E. Ross------------Fla.
E. D. Ward------------Texas
Drs. Alexander & Ragon--La.
Dr. Eugene Chowe-------Fla.
Dr. H. C. Dozier--------Fla.
Dr. Wm. P. Adamson----Fla.
Dr. W. H. Hope -------- S. C.
Dr. R. R. Kime ---------- Fla
Dr. W. H. Hendrick ------ Ga
Drs. Mason & Mason---- Ky
Dr. D. L. Yost -------- W. Va
Dr. C. Holtzclaw ------ Tenn
Dr. V. F. Dindsmore ---- Ga
Dr. M. R. Purnell ------ La
Dr. T. A. Neal ---------- Fla
Dr. H. J. Ray ---------- S. C
Dr. C. E. Shirey ---- Ala
Dr. F. R. Ivey -------- Miss
Dr. F. Clinton Moore -- Fla.
Dr. W. D. Roussell ------ La.
Dr. J. F. Mixon ------- Ga.
Dr. J. P. Lindsey ------ Tenn.
Dr. W. A. Burkhalter -- Miss.
Dr. Lee Jones ---------- Ala.
Dr. J. S. Helma -------- Fla.
Dr. H. W. Carter ---- N. C.
Dr. J. W. Seay -------- Miss.
Dr. ohn M. Kowe ------ La.
Dr. F. W. Parham ------ La.
Dr. M. D. Maddox ---- Ky.
Dr. E. R. Palmer ------ Ky.
Dr. W. W. Crawford -- Miss.
Dr. W. R. Owens ------ Ark.
Dr. W. R. Owens ------ Ark.
Dr. A. E. B. Alford ---- Ga.
Dr. E. S. Osbourne ---- Ga.
Dr. H. W. Terrell ------ Ga.
Dr. J. S. Hamilton ---- S. C.
Dr. A. M. Showalter ---- Va.
Dr. John Douglas ------ Ala.
Dr. W. T. Brockan ---- S. C.
Drs. Germillian & Germillian
------------------- La.
Dr. W. A. Stevens ------ Miss.
Dr. J. Blair Cross ---- Tenn.
Dr. W. H. Gray ------- Ky.

Dr. N. T. Yager -------- Ky.
Dr. P. Beekman ------ Miss.
Dr. Howard P. Deady -- Texas
Dr. A. K. Wilson ------ Fla.
Dr. Archie Griffin ------ Ga.
Dr. T. J. Deem ------ W. Va.
Dr. Ben C. Harris ---- Okla.
Drs. Chason & Chason - Ga.
Dr. Jno. F. Oncesner -- La.
Dr. J. H. McGinn ---- S. C.
Dr. W. O. Bricknell -- Tenn.
Dr. R. E. Adair -------- Ga.
Dr. W. L. Britt -------- Miss.
Dr. J. H. Johnson ---- Miss.
Dr. Robert B. Slocum --N. C.
Dr. P. E. Tanner ------ La.
Dr. E. G. Brown -------- Ga.
Dr. J. E. Herford ---- W. Va.
Dr. A. D. Stollinwerck -- Fla.
Dr. J. R. Polkinhorne -- Fla.
Dr. J. W. Powell ------ Ark.
Dr. Geo. W. Keene ------ Ga.
Dr. D. A. Haney ------ Ga.
Drs. Darrington & Swarze Miss
Dr. E. N. Passmore ---- Ala.
Dr. H. T. Harris ------ Ga.
Dr. T. K. Crovatt ------ Ga.
Dr. R. H. Rawls -------- Va.
Dr. E. R. Gaudy ------ La.
Dr. L. A. Abramson ---- La.
Dr. S. F. Martin -------- La.
Dr. N. A. McLeod ------ Miss.
Dr. C. G. Segars -------- Ala.
Dr. S. L. Brown ------ Texas
Dr. B. T. .Wise -------- Ga.
Dr. M. T. Summerlin -- Ga.
Dr. L. B. Morrison ------ S. C.
Dr. C. F. McLain ------ Ga.
Dr. E. B. Otts -------- La.
Dr. J. C. Culley ------ Miss.
Dr. W. R. McKinley -- Miss.
Dr. J. M. Bane -------- Ga.
Dr. A. W. Simpson ------ Ga.
Dr. A. C. Wade ------ Ga.
Dr. J. A. Brewer ------ Ala.
Dr. J. L. Wilson -------- La.
Dr. R. L. Huggins ------ Va.
Dr. J. W. Gilbert ----- Ky.
Dr. H. J. Danterive ---- La.
Dr. John Patterson ------ Ky.
Dr. Eugene Sample ---- Ala.
Dr. W. C. Howell -------- Ala.
Dr. J. W. Gray -------- Miss.
Dr. A. J. Carter ---- Miss.
Drs. Adams & Grave -- La.

